# Salivary Gland Extract from *Aedes aegypti* Improves Survival in Murine Polymicrobial Sepsis through Oxidative Mechanisms

**DOI:** 10.3390/cells7110182

**Published:** 2018-10-23

**Authors:** Rafaelli de Souza Gomes, Kely Campos Navegantes-Lima, Valter Vinícius Silva Monteiro, Ana Lígia de Brito Oliveira, Dávila Valentina Silva Rodrigues, Jordano Ferreira Reis, Antônio Rafael Quadros Gomes, Josiane Somariva Prophiro, Onilda Santos da Silva, Pedro Roosevelt Torres Romão, Jorge Eduardo Chang Estrada, Marta Chagas Monteiro

**Affiliations:** 1 Pharmaceutical Science Post-Graduation Program, Faculty of Pharmacy, Federal University of Pará, Pará 66075110, Brazil; rafaelli_gomes@hotmail.com (R.d.S.G.); kcnavegantes@gmail.com (K.C.N.-L.); any_015_@hotmail.com (A.L.d.B.O.); rafaelquadros13@hotmail.com (A.R.Q.G.); 2 Neuroscience and Cellular Biology Post Graduation Program, Institute of Biological Sciences, Federal University of Pará, Pará 66075110, Brazil; jorgechang01@gmail.com; 3 Laboratory of Inflammation and Pain, Department of Pharmacology, Ribeirão Preto Medical School, University of São Paulo, São Paulo 14049900, Brazil; valterv@live.com; 4 School of Pharmacy, Health Science Institute, Federal University of Pará, Pará 66075110, Brazil; davilavrodrigues@gmail.com (D.V.S.R.); jordanoreis@outlook.com (J.F.R.); 5 Pharmaceutical Innovation Post-Graduation Program, Institute of Health Sciences, Federal University of Pará, Pará 66075110, Brazil; 6 Department of Biological Sciences and Health, University of Southern Santa Catarina, Santa Catarina 88704900, Brazil; josiane.prophiro@hotmail.com; 7 Department of Microbiology, Immunology and Parasitology, Basic Health Sciences Institute, Federal University of Rio Grande do Sul, Rio Grande do Sul 90040060, Brazil; onilda.santos@gmail.com; 8 Laboratory of Cellular and Molecular Immunology, Department of Basic Sciences, Federal University of Health Sciences of Porto Alegre, 90050170, Brazil; pedror@ufcspa.edu.br

**Keywords:** *Aedes aegypti*, salivary gland extract, saliva, sepsis, CLP model, oxidative stress

## Abstract

Sepsis is a systemic disease with life-threatening potential and is characterized by a dysregulated immune response from the host to an infection. The organic dysfunction in sepsis is associated with the production of inflammatory cascades and oxidative stress. Previous studies showed that *Aedes aegypti* saliva has anti-inflammatory, immunomodulatory, and antioxidant properties. Considering inflammation and the role of oxidative stress in sepsis, we investigated the effect of pretreatment with salivary gland extract (SGE) from *Ae. aegypti* in the induction of inflammatory and oxidative processes in a murine cecum ligation and puncture (CLP) model. Here, we evaluated animal survival for 16 days, as well as bacterial load, leukocyte migration, and oxidative parameters. We found that the SGE pretreatment improved the survival of septic mice, reduced bacterial load and neutrophil influx, and increased nitric oxide (NO) production in the peritoneal cavity. With regard to oxidative status, SGE increased antioxidant defenses as measured by Trolox equivalent antioxidant capacity (TEAC) and glutathione (GSH), while reducing levels of the oxidative stress marker malondialdehyde (MDA). Altogether, these data suggest that SGE plays a protective role in septic animals, contributing to oxidative and inflammatory balance during sepsis. Therefore, *Ae. aegypti* SGE is a potential source for new therapeutic molecule(s) in polymicrobial sepsis, and this effect seems to be mediated by the control of inflammation and oxidative damage.

## 1. Introduction

Sepsis is a potentially fatal form of organ dysfunction caused by a dysregulated immune response to an infection [1,2]. It is the second leading cause of death of patients in intensive care units (ICUs) worldwide. One of the main factors contributing to this result is the use of inadequate or late therapy [3,4]. Over the past few years, studies have shown that patients with sepsis have marked oxidative stress and inflammatory processes which may lead to cell damage [5]. In animals, the model of cecal ligation and puncture (CLP) mimics the conditions of human sepsis, and involves polymicrobial infection with a dysregulated immune response and oxidative process [6]. Thus, this animal model is excellent for evaluating new therapies that can be used in sepsis, since the first line of sepsis treatment is empirical (using broad-spectrum antimicrobials) but is not sufficient for infection control due to the unstable clinical status of the patient [7,8,9].

In recent years, substances with antioxidant and immunomodulatory actions have been tested in the CLP model in order to generate a balance between inflammatory and oxidative processes, leading to increased survival of septic animals [10,11]. Therefore, new therapies with saliva of hematophagous insects are excellent candidates, since their salivary components, rich sources of bioactive substances, have different pharmacological effects that can modulate immunological, anticoagulant, and oxidative systems that are totally unbalanced in the organism, leading to homeostasis and to the clinical improvement of the host [12,13,14,15,16].

The salivary glands of blood-feeding arthropods secrete more than 55 bioactive molecules during the blood feeding, such as carrier-type proteins including Aegyptin, the D7 family, protease inhibitors (i.e., serpins, Kazal-containing peptide, and cystatin), serine proteases, nucleotidases, immunity-related proteins, and mucins, among others [13,14]. The saliva of the *Aedes aegypti* mosquito, the primary vector of several diseases such as yellow fever, dengue, and Zika [15,16], has several salivary components with described biological properties, including antiplatelet, vasodilator, anticoagulant, anti-inflammatory, immunomodulatory, and antioxidant activities [12,13,17]. Focusing on immunosuppressive action of salivary components, Sales-Campos et al. (2015) showed that *Ae. aegypti* salivary gland extract (SGE) and its fractioned components were able to reduce the severity and clinically improved experimental colitis, without causing any cytotoxicity to RAW 264.7 macrophages in vitro [12]. Therefore, we question whether the *Ae. aegypti* SGE could be used in prophylactic or therapeutic treatment in pathologies of intense inflammatory and oxidative processes such as sepsis. Thus, this study aimed to evaluate the effect of the pretreatment of SGE from *Ae. aegypti* in the induction of inflammatory and oxidative processes in a murine CLP model.

## 2. Material and Methods

### 2.1. Ethics Statement

This study was carried out in strict accordance with the recommendations of the Guide for the Care and Use of Laboratory Animals of the Brazilian National Council of Animal Experimentation (http://www.sbcal.org.br/) and the NIH Guidelines for the Care and Use of Laboratory Animals. The institutional Committee for Animal Ethics of Federal University of Pará/UFPA (CEUA, Protocol: 5919210516) approved all the procedures used in this study.

### 2.2. Mice

Seven-to-eight-week-old male Swiss mice were used in all experiments. Animals were obtained from the Animal Facility of the Federal University of Pará and were kept in a 12:12-h light/dark cycle (lights on at 07:00), in groups of five mice per cage, with food and water ad libitum with a 3-day period of housing acclimation before CLP. The outbred mice were used to preserve the heterogeneity found in septic patients as described by Ferreira et al. (2017) [18]. 

### 2.3. Aedes aegypti Salivary Gland Extraction

SGE from sugar-fed 3–7-day-old female *Ae. aegypti* mosquitoes (Rio Grande do Sul, Brazil) were dissected in water containing 0.1% (*w*/*v*) bovine serum albumin. To achieve complete disruption, the glands were freeze-thawed and vortexed. The osmolarity was adjusted by adding 10× phosphate-buffered saline (PBS), as described by Monteiro et al. (2005) [19]. The protein concentration was determined using NanoDrop 2000 (Thermo Fisher Scientific, Wilmington, DE, USA) and aliquots were kept at −80 °C until the time of use.

### 2.4. Experimental Design and CLP Model 

**Figure 1:**
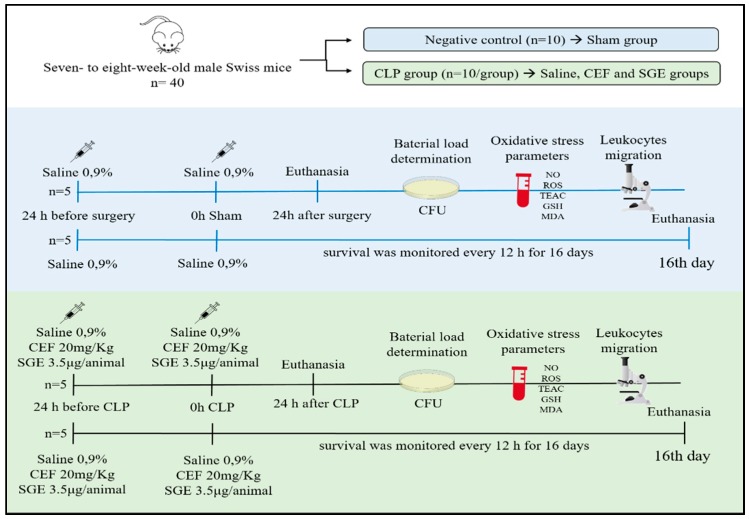
Experimental protocol of the CLP model and pre-treatments. CLP: cecal ligation and puncture; CEF: ceftriaxone; CFU: colony-forming unit; ROS: reactive oxygen species; SGE: salivary gland extract; TEAC: Trolox equivalent antioxidant capacity.

Mice were randomly allocated into four groups (*n* = 10 per group) and all animals received saline, ceftriaxone, or SGE, intraperitoneally, 24 h before and at time zero of CLP induction. Group I (sham-operated mice) underwent a similar procedure to mice of the other groups, but the caecum was not ligated or punctured. This group was pretreated with 0.9% saline and acted as a negative control group. Animals from the other three groups received two intraperitoneal (i.p.) doses of saline, ceftriaxone (CEF—20 mg/kg), or salivary gland extract (SGE—3.5 μg/animal) 24 h beforehand and at time zero, and then the CLP was performed (Figure 1). The polymicrobial sepsis was induced using the CLP method according to the experimental protocol described above by d’Acampora and Locks (2014) and Rittirsch et al. (2014), following some adaptations [20,21]. Briefly, mice were anaesthetized by intraperitoneal (i.p.) administration of 200 μL of ketamine (100 mg/kg) and xylazine (10 mg/kg) solution. Under aseptic conditions, a 1- to 2-cm midline laparotomy was performed to allow the exposure of the caecum. The caecum was ligated with a 3-0 silk suture at its base, below the ileocecal valve, without causing bowel obstruction. Thus, the caecum was punctured one single time (crossing both intestine walls) with a 22 gauge needle to induce the degree of sublethal lethality. Then, the caecum was squeezed, and a “controlled” amount of cecal content was released through the punctures. The caecum was placed back in the abdominal cavity, and the peritoneal wall and skin incision were sutured. Sham-operated mice underwent a similar procedure, but the caecum was not ligated or punctured. The animals received sterile saline (1 mL) subcutaneously immediately after the surgery, which is essential for producing the hyperdynamic phase of sepsis during earlier stages in this experimental model. After the procedure, the animals received doses of buprenorphine (0.05 mg/kg) for postoperative analgesia every 6 h for at least 2 days. Chow pellets were kept inside the cage after CLP procedure for the entire period of the experiment.

### 2.5. Survival and Weight Analysis

After the CLP procedure survival was monitored every 12 h for 16 days in each group and mice were weighted for the weight curve. Differences in survival and weight were analyzed using Prism 6 software (Graph Pad Software, La Jolla, CA, USA). At this time, mice that showed signs of imminent death (i.e., inability to maintain upright position/ataxia/tremor and/or agonal breathing) were euthanized by ketamine/xylazine (>100/10 mg/kg, sc) overdose. At the end of 16 days, live mice were euthanized. The number of animals for survival curves was 5–10 per each group. 

### 2.6. Sample Collection for Cell Migration and Oxidative Evaluation

Inflammatory and oxidative parameters were determined at 12 or 24 h after CLP procedure. For this, the animals were euthanized and the peritoneal cavity cells were harvested with 3 mL of PBS containing 1 mM EDTA. The volumes recovered were similar in all experimental groups and 95% of the injected volumes were recovered. Total counts were performed in a cell counter and differential cell counts (200 cells total) were carried out on cytocentrifuge slides stained with panoptic dye. The results were presented as the number of neutrophils per cavity. The blood was collected for leucogram and liver, spleen, lung, and heart were obtained, homogenated, and kept at −80 °C until analysis.

### 2.7. Bacterial Load Determination

Blood, peritoneal lavage fluid, and whole organ (liver, spleen, lung, and heart) were placed in 900 µL of sterile PBS and then mechanically disrupted by macerating the organ with a sterile Pasteur pipette and then vortexing vigorously. For determination of colony-forming units (CFUs), aliquots (100 µL) of supernatant from disrupted organs were diluted (1:100). Then, 10 μL of supernatant were plated on Müller Hinton Agar and incubated at 37 °C for 24 h. The colonies were counted and expressed in CFU/gram tissue. Bacterial load was determined in all tissues of all five mice for group and the data represents the means ± SD of five mice. 

### 2.8. Determination of Nitric Oxide (NO) Production

The nitrite (NO_2_) concentration was determined by the Griess method [22]. Briefly, 100 µL of the samples was incubated with an equal volume of the Griess reagent for 10 min at room temperature. The absorbance was measured on a plate scanner (Spectra Max 250; Molecular Devices, Menlo Park, CA, USA) at 550 nm. The nitrite concentration was determined using a standard curve generated using sodium nitrate (NaNO_2_). Nitrite production is expressed per µM [23].

### 2.9. Measurement of Reactive Oxygen Species (ROS) Production

ROS production was performed according to Ferreira-Cravo et al. (2007) [24] using 2’,7’-Dichlorodihydrofluorescein diacetate (H_2_DCF-DA, Sigma-Aldrich, Saint Louis, MO, USA). The H_2_DCF-DA is a cell-permeable non-fluorescent probe that is hydrolyzed enzymatically by intracellular esterases to form the intermediate to non-fluorescent 2,7-dichlorodihydrofluorescein (DCFH) that reacts with various ROS (including H_2_O_2_, OH•, and O_2_•^−^) and also by RNS (•NO and ONOO^−^) to form 2’,7’-dichlorofluorescein (DCF), a highly fluorescent product [25]. Thus, peritoneal macrophages were exposed to 40 μM tert-butylhydroperoxide (t-BHP) (Sigma-Aldrich, Saint Louis, MO, USA) or saline for 30 min at 5% CO_2_ at 37 °C [22]. t-BHP is an organic peroxide widely used in a variety of oxidation processes, and was used here as a positive control [24]. Twenty minutes before the end the exposure with t-BHP, 10 μM H_2_DCF-DA was added to the suspension and incubated for 30 min at 37 °C. Immediately, the DCF fluorescence intensity was measured by a fluorescence microplate reader (Victor 2, Perkin Elmer, Waltham, MA, USA) every 5 min for 30 min at an excitation wavelength of 488 nm with a 530-nm emission filter [24]. Background fluorescence was determined before the addition of H_2_DCF-DA. The amount of intracellular ROS and RNS was expressed in terms of fluorescence intensity. 

### 2.10. Total Evaluation of Trolox Equivalent Antioxidant Capacity (TEAC)

The total antioxidant status (TAS) is a sensitive and reliable marker to detect in vivo oxidative stress changes that may not be detectable through the measurement of a single specific antioxidant. The TAS was evaluated by Trolox ((±)-6-Hydroxy-2,5,7,8-tetramethylchromane-2-carboxylic acid; Sigma-Aldrich) equivalent antioxidant capacity assay from samples of the serum and peritoneal lavage. In this assay, 2,2-azino-bis (3-ethylbenzothiazoline, 6-sulfonate) (ABTS) is incubated with potassium persulfate (K_2_S_2_O_8_; Sigma-Aldrich, Saint Louis, MO, USA) to produce ABTS•^+^, which is a green/blue chromophore. Antioxidants present in the sample cause a reduction in absorption proportional to their concentration. The antioxidant capacities of the samples are expressed as TEAC using a calibration curve plotted with different amounts of Trolox, and their absorbance measured at 740 nm [26,27]. Data were expressed as µmol/L. 

### 2.11. Glutathione (GSH) Levels

Determination of GSH levels were determined in the serum, peritoneal lavage, and organ homogenate according to the method described by Ellman (1951) [28]. Intracellular GSH was based on the ability of GSH to reduce 5,5-dithiobis-2-nitrobenzoic acid (DTNB) to nitrobenzoic acid (TNB), which was quantified by spectrophotometry at 412 nm, and GSH concentrations were expressed in µmol/mL. This assay was adapted for use in a microtiter plate using a microplate spectrophotometer system, Spectra MAX 250 (Molecular Devices, Union City, CA, USA) [17].

### 2.12. Determination of Lipid Peroxidation

Lipid peroxidation was measured by quantifying MDA in the serum, peritoneal lavage and organ homogenate, as an indicator of oxidative stress, using the thiobarbituric acid-reactive substances (TBARS) assay. The technical procedure was performed according to the protocol proposed by Kohn and Liversedge (1944), adapted by Percario et al. (1994) [29,30]. Briefly, lipoproteins were precipitated by the addition of samples to 0.05 M trichloroacetic acid (TCA) and 0.67% thiobarbituric acid (TBA; Sigma-Aldrich, Saint Louis, MO, USA) in 2 M sodium sulfate. The union of lipid peroxide and TBA was performed by heating in a water bath for 90 min. The chromogen formed was extracted in n-butanol, which was measured at a wavelength of 535 nm. Lipid peroxidation was expressed as nanomoles of MDA per liter. 

### 2.13. Statistical Analysis

Data are expressed as the mean ± SD values. Statistically significant differences between groups were determined using analysis of variance (ANOVA) followed by the Tukey multiple comparison tests. In all cases, the significance level adopted was 5% (*p* < 0.05). Data of survival was analyzed by employing Kaplan–Meier analysis.

## 3. Results

### 3.1. SGE Protected Mice from Sepsis-Induced Lethality

**Figure 2:**
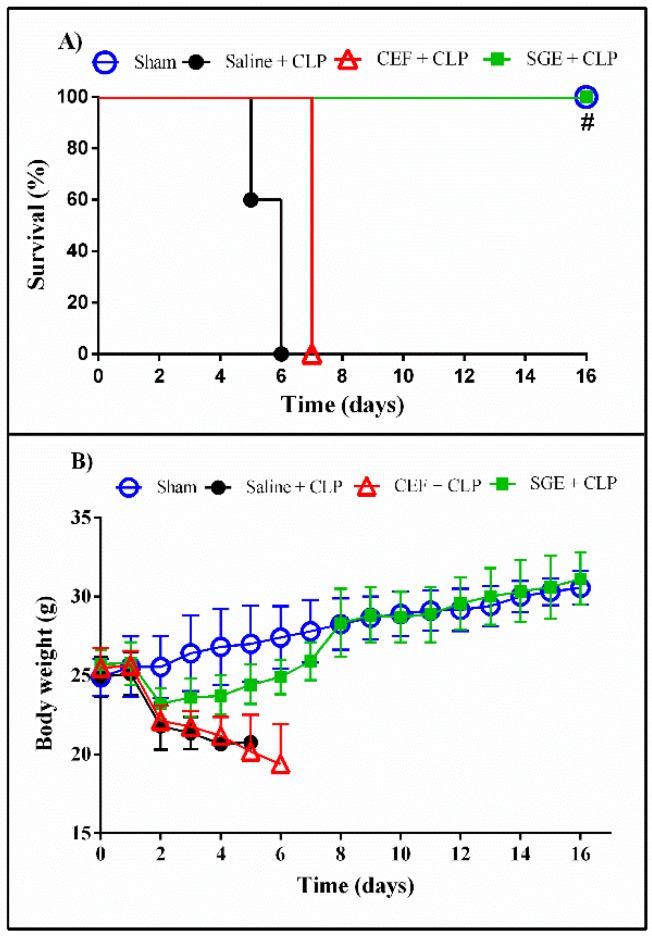
Effect of SGE on survival rate (%) and body weight (g) of septic mice within 16 days. (**A**) Survival of CLP animals pretreated with SGE (3.5 µg/animal), CEF (20 mg/kg), or saline. (**B**) Body weight during time evaluated for survival. Data presented as mean ± SD (*n* = 5). Kaplan–Meier analysis (# *p* < 0.005 CLP versus CLP + saline). CEF: ceftriaxone; CLP: cecal ligation and puncture SGE: salivary gland extract.

We initially verified whether the SGE were able to improve the survival rate and ameliorate the weight loss of CLP-induced animals. Figure 2 shows that saline-pretreated CLP animals died up to six days after induction of sepsis, while SGE-pretreated (3.5 μg/animal) CLP animals survived at least until day 16. Ceftriaxone pretreatment was used as the control for prophylactic antimicrobial therapy; deaths occurred in CLP animals pretreated with CEF up until the seventh day. In addition, body weight was monitored in order to relate the general physical conditions of animals, as shown in Figure 2B. All animals showed a decrease in body weight after induction of sepsis until the fourth day. However, after this period the SGE-pretreated CLP animals recovered their body weight.

### 3.2. SGE Decreased Bacterial Load in Septic Animals.

The bacterial load was evaluated to access the progression of the infection. Table 1 shows that saline-pretreated CLP animals had high bacterial load, while CEF or SGE-treated CLP animals had a lower number of bacteria at 12 h after induction of sepsis in the peritoneum, blood, and in all tissues evaluated. Moreover, within 24 h, the bacterial load was not detected in most evaluated tissues, showing the antimicrobial and protective effects of SGE in this sepsis model.

### 3.3. SGE Altered Leukocyte Migration

**Figure 3:**
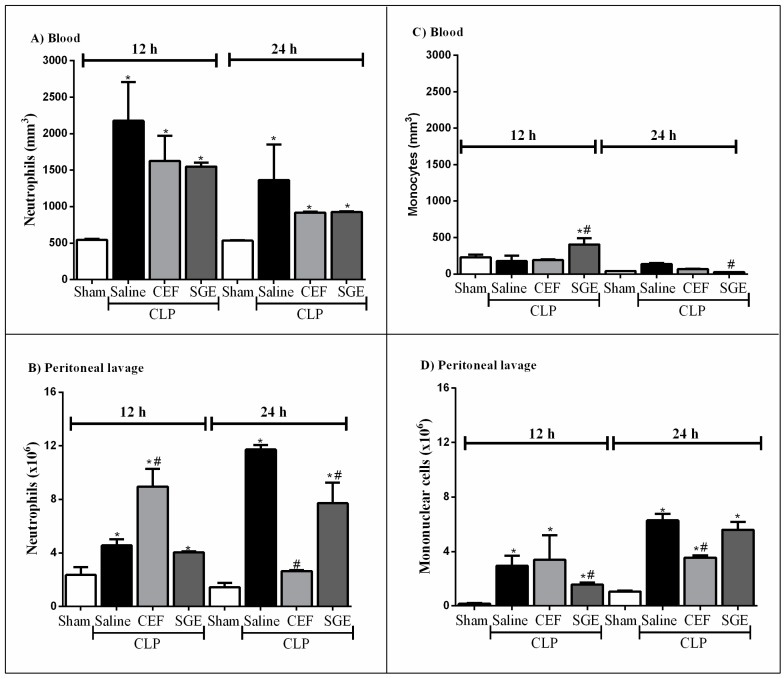
Effect of SGE on the number of circulating cells and migration to the peritoneal cavity in animals with sepsis. (**A**) Neutrophils in the blood. (**B**) neutrophils in the peritoneal cavity. (**C**) monocytes in the blood. (**D**) Monocytes in the peritoneal cavity. Total cell counts in the peritoneal cavity and blood were determined 12 h and 24 h after CLP. The results were expressed as the mean ± SD (five animals/group). * *p* < 0.05 compared to the sham control group; # *p* < 0.05 compared to the saline control group. CLP: cecal ligation and puncture; SGE: salivary gland extract; CEF: ceftriaxone.

The migration of leukocytes to sites of infection is an essential step for bacteria elimination and survival during sepsis. Figure 3 shows an increase in the number of neutrophils in the blood (panel A) and peritoneal cavity (panel B) 12 and 24 h after induction of sepsis. The numbers of neutrophils in the blood of septic animals were similar between treated groups and higher in relation to the sham group (panel A). At 12 h after CLP induction, the numbers of neutrophils in the peritoneal cavity of animals were significantly higher in CEF-treated groups as compared to the saline- or SGE-treated groups. However, at 24 h post CLP, the treatment with SGE (and with CEF) was able to decrease the neutrophil influx to the peritoneal cavity (panel B). 

On the other hand, the pre-treatment with SGE increased the number of monocytes in the blood of septic animals at 12 h after CLP, with a significant decrease 24 h after sepsis with respect to animals from the saline group (Figure 3C). 

With respect to mononuclear cells, the saline-pretreated septic animals showed a significant increase in the number of these cells in the peritoneal fluid after 12 h, with a peak of influx at 24 h compared to sham groups (Figure 3D). The pre-treatment with SGE reduced the mononuclear cell influx at 12 h but not at 24 h after CLP induction (panel D), while the pretreatment with CEF inhibited these cells only within 24 h, as compared to saline-pretreated CLP group (Figure 3D). In summary, SGE significantly altered the influx of neutrophils into the peritoneal cavity.

### 3.4. SGE Modulates NO and ROS Production in Septic Mice

**Figure 4:**
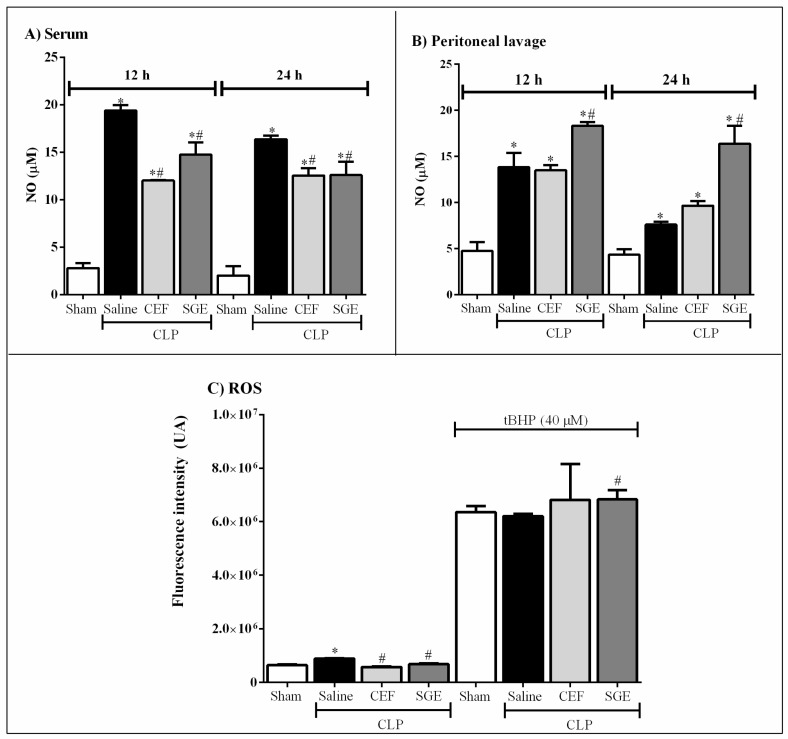
Effect of SGE on NO and ROS production in septic mice. (**A**) NO levels in serum 12 h and 24 h after CLP; (**B**) NO levels in the peritoneal cavity 12 h and 24 h after CLP; (**C**) ROS production by peritoneal macrophages stimulated or not with 40 μM t-BHP. The results were expressed as the mean ± SD (five animals/group). * *p* < 0.05 compared to sham control group; # *p* < 0.05 compared to the saline control group. CLP: cecal ligation and puncture; SGE: salivary gland extract; CEF: ceftriaxone; NO: nitric oxide; ROS: reactive oxygen species.

To access the oxidative status in septic animals, the production of NO and intracellular ROS was analyzed. Saline-pretreated CLP animals presented increased levels of NO in the serum (Figure 4 panel A) and on peritoneal lavage (panel B) at 12 h, remaining at high levels at 24 h compared to sham animals (Figure 4). The pretreatment with CEF or SGE lightly reduced the NO production after 12 and 24 h in blood from animals with sepsis, as compared to saline-pretreated animals (Figure 4A). The pretreatment with SGE increased the NO levels induced by infection in the peritoneal cavity, while pretreatment with CEF did not change the production of this mediator at all evaluated times compared to saline-pretreated animals (Figure 4B). Figure 4C shows that in vitro the peritoneal cells collected from septic animals, regardless of the group (saline, CEF, or SGE-treated), produced significant levels of ROS compared to the sham group. However, this production was lower in the SGE or CEF-treated group in relation to the sham group. In the presence of t-BHP, cells from the SGE-pretreated CLP animals produced higher amounts of ROS compared to saline-pretreated septic or sham animals (Figure 4C). These data suggest that the cells of SGE-pretreated animals can produce high amounts of ROS and reactive nitrogen species (RNS) when stimulated in vitro. However, with an inflammatory/infectious focus, these cells may produce moderate amounts of ROS and RNS, avoiding severe oxidative damage in the tissue but allowing the elimination of the infectious agent.

### 3.5. SGE Increased Antioxidant Factors

**Figure 5:**
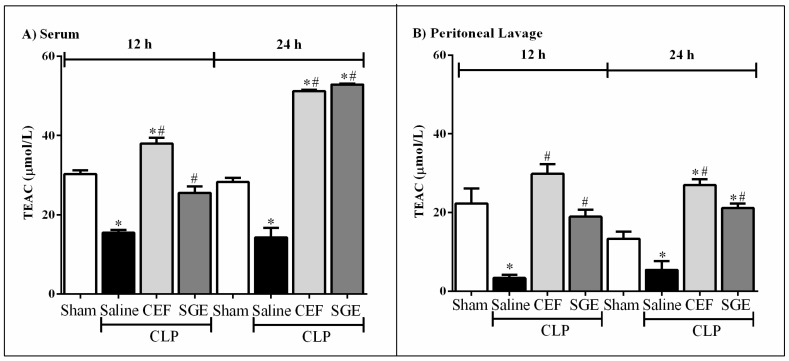
Effect of SGE on antioxidant activity in septic mice. (**A**) TEAC in serum 12 h and 24 h after CLP; (**B**) TEAC in the peritoneal cavity 12 h and 24 h after CLP. The results were expressed as the mean ± SD (five animals/group). * *p* < 0.05 compared to the sham control group; # *p* < 0.05 compared to the saline control group. CLP: cecal ligation and puncture; SGE: salivary gland extract; CEF: ceftriaxone; TEAC: Trolox equivalent antioxidant capacity.

Trolox equivalent antioxidant capacity (TEAC) assay was conducted to evaluate the antioxidant parameters in septic animals. Figure 5 shows that saline-pretreated septic animals had a marked reduction in the antioxidant capacity in the serum and peritoneal cavity at the times evaluated as compared to sham animals (Figure 5A,B, respectively). On the other hand, pretreatment with SGE or CEF was able to increase the serum TEAC levels, especially at 24 h, as compared to saline-pretreated CLP groups (Figure 5A). In the peritoneal cavity, the pre-treatment with SGE or CEF also elevated the total antioxidant levels compared to sham or saline-pretreated CLP animals (Figure 5B).

### 3.6. SGE Increased GSH Levels 

**Figure 6:**
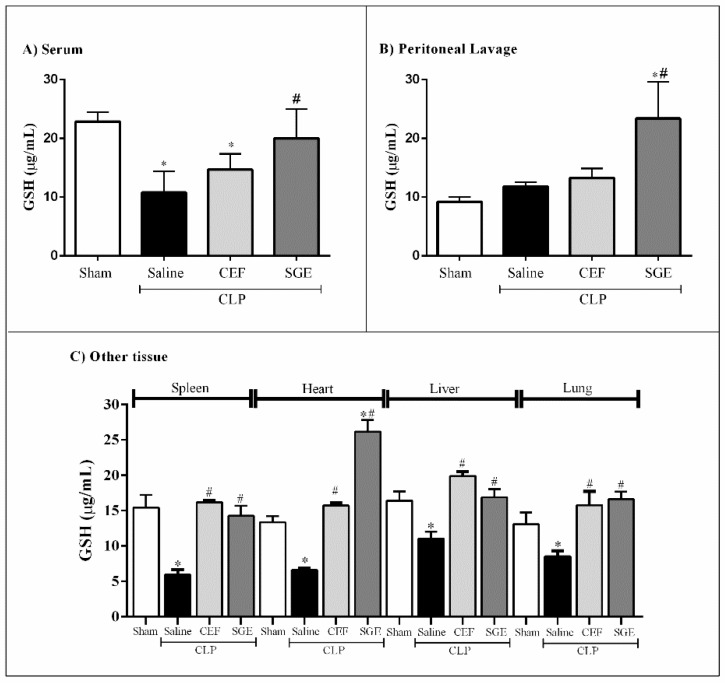
Effect of SGE on GSH levels in septic mice. (**A**) GSH levels in serum 12 h and 24 h after CLP. (**B**) GSH levels in peritoneal cavity 12 h and 24 h after CLP. (**C**) GSH levels in other tissue after 12 h CLP. The results were expressed as the mean ± SD (five animals/group). * *p* < 0.05 compared to the sham control group; # *p* < 0.05 compared to saline-pretreated sepsis group. CLP: cecal ligation and puncture; SGE: salivary gland extract; CEF: ceftriaxone; GSH: glutathione.

As TEAC was increased in SGE pretreated septic animals, GSH levels were also analyzed, since it is an important endogenous antioxidant able to neutralize ROS. Figure 6 shows that the induction of sepsis led to a significant decrease in GSH levels in the serum, spleen, heart, liver, and lung of animals (Figure 6A,C, respectively), without any significant alteration in peritoneal lavage compared to sham animals (Figure 6B). However, the pretreatment with SGE was able to restore the GSH levels in the blood and in most tissues evaluated, and caused a greater increase in heart and peritoneal lavage compared to the sham or saline-pretreated CLP animals (Figure 6A–C). 

### 3.7. SGE Reduced MDA Levels in Septic Animals

**Figure 7:**
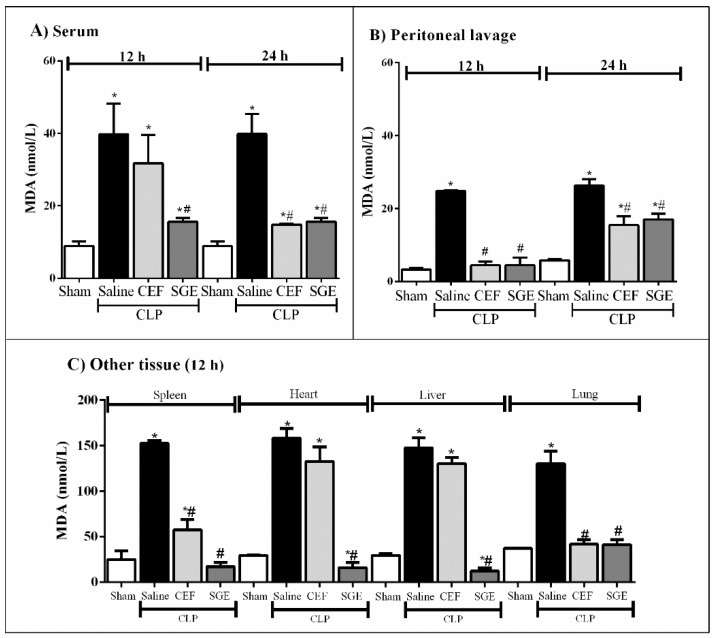
Effect of SGE on lipid peroxidation products in septic mice. (**A**) MDA levels in serum at 12 and 24 h after CLP. (**B**) MDA levels in the peritoneal cavity 12 and 24 h after CLP. (**C**) MDA levels in other tissue 12 h after CLP. The results are expressed as the mean ± SD (five animals/group). * *p* < 0.05 compared to the sham group; # *p* < 0.05 compared to saline-pretreated septic group. CLP: cecal ligation and puncture; SGE: salivary gland extract; CEF: ceftriaxone; MDA: malondialdehyde.

MDA levels were used as a marker of lipid peroxidation caused by oxidative imbalance. Figure 7 shows that saline-pretreated septic animals presented elevated MDA levels in serum, peritoneal lavage, and in all tissues analyzed at 12 and 24 h as compared to the sham group. The pretreatment with CEF reduced the lipid peroxidation in peritoneal lavage, spleen and lung at 12 h after CLP. On the other hand, SGE-pretreated CLP animals showed a reduction in MDA detection in the blood, peritoneal lavage, and all tissues evaluated as compared to the saline-pretreated group (Figure 7A–C). Thus, these data suggest that SGE controls the intense oxidative process in the inflammatory focus, while decreasing the bacterial load during the polymicrobial sepsis.

## 4. Discussion

Our study showed the prophylactic effect of *Ae. aegypti’s* SGE in a model of sepsis induced by CLP through increased survival, reduced bacterial load, and modulation of leukocyte influx and oxidative status in septic animals. In addition, it was the first study to show that the SGE has an excellent antioxidant action during sepsis, being able to increase antioxidant defense based on GSH and total antioxidant capacity, and reduce the oxidative damage mediated by ROS and lipid peroxidation.

Therefore, we showed for the first time that SGE of *Ae. aegypti* protects mice against oxidative stress in a CLP model, which is considered the gold standard sepsis model [31] and most closely represents the clinical scenario with progression of sepsis [32]. In this model, the severity of sepsis can be adjusted by increasing the needle puncture size, number of punctures, or length of cecal ligation [31,33,34]. Thus, we performed a moderate CLP (50%, two punctures with a 22G needle) so that the animals died in the 6 days after CLP [21,35]. In our studies, animals of the saline group died within 6 days, those in the CEF group died within 7 days, while those pretreated with SGE survived until the end of 16th day, recovered their body weight, and were shown be healthy. 

In recent years, antibiotic prophylaxis has been implemented worldwide to reduce the risks of secondary hospitalizations and increased mortality due to infectious complications in urinary tract infections, urosepsis, cirrhosis, caesarean sections, biopsy procedures and others [21,35]. Therefore, in surgical practice, antibiotic prophylaxis is a standard practice in many procedures, including procedures to prevent post-biopsy infections. In this sense, several medical associations recommend prophylaxis with a single agent such as cephalosporins (drug of choice), fluoroquinolones, trimethoprim-sulfamethoxazole, or aminoglycosides (alternatives). However, in general, prophylactic treatment is short-term and generally maintained until the 48th postoperative hour [21,35]. Thus, in our study, ceftriaxone, a broad-spectrum cephalosporin with action on gram-positive and gram-negative bacteria, was used as a reference drug (positive control) for prophylactic treatment. Although the treatment with ceftriaxone caused a significant decrease in bacterial load and in inflammatory and oxidative parameters in most tested tissues of septic animals, it was not able to increase the survival of septic animals. In this context, the decline in neutrophils and mononuclear cells induced by pretreatment with ceftriaxone within 24 h may be the result of its antibacterial activity leading to decreased levels of cytokines and chemokines in tissues, as reported recently by Patel et al. (2018) [36]. Regarding oxidative parameters, ceftriaxone showed a moderate antioxidant effect, which can be attributed to the low concentrations of this drug in the tissue due to its extensive binding to plasma proteins [37].

The CLP procedure promotes ischemia in the region of the cecum, as well as bacterial translocation favoring the polymicrobial infection [38]. In the CLP model, the predominant bacterial strains in the abdominal cavity are *Escherichia coli*, *Enterococcus faecalis*, *Proteus mirabilis*, *Klebsiella pneumoniae*, and *Enterobacter agglomerans* [21,38]. Thereby, these bacteria can be transported via lymphatics and blood to vital organs, causing an intense systemic inflammatory response [39]. According to Hyde et al. (1990), in the various stages of sepsis in the CLP model there are no differences in the three predominant types of bacteria isolated from the cecum (anaerobes, gram-positive bacteria, and aerobic coliforms). In addition, correlation tests showed that bacteria isolated from tissues such as the liver and spleen reflected the same type of bacteria found in the circulation and in cecum ligation [40]. Thus, the control of bacterial burden has an important impact on mortality in sepsis [41]. In this regard, many components from the SGE of *Ae. aegipty* can also modulate innate and adaptive immunity through D7, sialokinin, Aegyptin, and components with antimicrobial action such as lysozymes and AMPs [42,43,44]. Moreover, antioxidant components such as superoxide dismutase (SOD) and glutathione s-transferase (GST) can also contribute to the reduction of oxidative stress and increased survival rate [45]. 

Our data showed that the pretreatment with SGE reduced the bacterial load in most evaluated tissues of septic animals. The in vitro antimicrobial effect of SGE of *Ae. aegypti* was reported [46]. In fact, the salivary glands of mosquitoes produce some antimicrobial polypeptides. For example, in SGE of *Ae. aegypti* the abundant expression of the lysozyme gene (*AAEL009670*) was found in the proximal regions of lateral lobes, conferring a bacteriolytic factor protecting the mosquito against invasion and bacterial growth during the ingestion of sugars in the wild [14,46,47]. Moreover, according to Sim et al. [48], *Ae. aegypti* SGE has eight antimicrobial peptides (AMPs such as cecropin, defensin A1, and gambicin [14,47]) that can interact with bacterial surfaces, resulting in their elimination by mechanisms that lead to lysis, disruption, or membrane perturbations [44,49]. These components exhibit a wide spectrum of antimicrobial activity against gram-negative and gram-positive bacteria, and gambicin also has an effect against filamentous fungi [50,51]. Moreover, another studies reported other components with antimicrobial effects such as gram-negative binding proteins (GNBP), which bind to gram-negative bacteria and putative salivary peptides with an HHH domain [14,43,52].

Polymicrobial sepsis is also characterized by increased inflammatory response in the early phase, with high leukocyte migration within the first 24 h, increased production of pro-inflammatory mediators such leukotrienes (LTs) and TNF-α, and an increase ROS that contributes to cell and tissue injury, resulting in mortality [53]. In the CLP model, during the inflammatory process there is an increase in neutrophil and monocyte recruitment from the vascular lumen to the inflammatory site [54]. Neutrophil influx is important for the control of bacterial growth and consequent dissemination [55,56]. In the early-arriving phase, there is an increase in LT production, mainly leukotriene B4 (LTB4) [57,58]. In wild-type mice subjected to CLP, high levels of LTB4 have been associated with an increase in leukocyte recruitment [35]. According to Calvo and collaborators [42], the D7 protein found in SGE from *Ae. aegypti* binds to LTB4 and decreases neutrophil and macrophage recruitment into the inflammatory site. Previously, our group also reported that the saliva of another blood-sucking insect, *Lutzomyia longipalpis* (a *Leishmania* vector), was also able to modulate the cellular response (neutrophils, macrophages and T lymphocytes) by mechanisms dependent on TNF-α and LTB4 [19,56]. Therefore, these data suggest that the SGE of *Ae. aegypti* may act by inhibiting LTB4 and TNF-α, leading to a reduction in the influx of macrophages and neutrophils to the inflammatory focus.

Previous studies have already reported the immunomodulatory and hemostatic effects of *Ae. aegypti’s* SGE in vitro due its salivary components [59], which include the D7 protein (37-kDa salivary gland allergen Aed a 2 precursor), and the N-terminal domain, which binds the biogenic amines of molecule effectors such as LTC4, LTD4, and LTE4 in cases of allergy [42,43]. Sialokinin can modulate Th1/Th2 cytokine production [44,60], as well as adenosine (a vasodilator and inhibitor of platelet aggregation) which can reduce pain during blood repayment of the mosquito [14]. Aegyptin has anticoagulant action by specifically binding to collagen and preventing its interaction with platelet glycoprotein VI, α2β1 integrin, and the Von Willebrand factor [61]. There are few studies about the effect of *Ae. aegypti’s* SGE in animal disease models [12], but none have shown its antioxidant activity in vivo. In this regard, Sales-Campos et al. [12] have also shown in an inflammatory bowel disease model that SGE of *Ae. aegypti* (5 μg/animal) reduced inflammatory infiltrate in the intestine between 9 and 24 days after induction of colitis, reduced levels of pro-inflammatory cytokines, and improved the clinical picture of the disease. SGE of *Ae. aegypti* also reduced T cell recruitment and increased levels of IL-10, suggesting an anti-inflammatory effect during West Nile virus infection in mice [62]. Saliva of other hematophagous organisms can alter the influx of cells into the infected tissue, as reported by Monteiro et al. [63], who showed that the SGE from *Lutzomyia longipalpis* increased the recruitment of leukocytes, mainly macrophages, into the peritoneal cavity until at least 7 days after *Leishmania* infection. 

Regarding the oxidative process, some *Ae. aegypti* salivary components are able to modulate these parameters during blood digestion. Thereby, antioxidants such as glutathione peroxidase (GPx), GST, catalase, and GSH can neutralize pro-oxidant molecules toxic to the mosquito [64,65]. In addition, other studies [17,45] also showed that *Ae. aegypti* salivary antigen-5/CAP is a superoxide dismutase that bind Cu^2+^ and scavenges O^2−^, blocking the oxidative neutrophil burst induced by phorbol 12-myristate13-acetate (PMA). 

In the inflammatory site, neutrophils and macrophages destroy pathogens through phagocytosis and ROS production, involving superoxide anions (O^2-^), hydrogen peroxide (H_2_O_2_), HOCl and hydroxyl radicals (OH^−^), and RNS (NO and peroxynitrite), as well as proteolytic enzymes and pro-inflammatory cytokines [56,57,66,67]. However, the uncontrolled activation of these cells may lead to oxidative stress resulting from an intense inflammatory process and dysfunction of the endothelial cells which can lead to a multiple organ failure in sepsis [68], as well as a metabolic imbalance in the organism and depletion of antioxidant factors such as SOD and GSH [69]. As a consequence of this imbalance, the reactive species cause a series of reversible and irreversible toxic modifications in the biomolecules, such as protein carbonylation and lipid peroxidation, leading to damage in their own cells, including the endothelium [70]. 

NO is one of the major RNS in the human system [71]. NO acts as a potent vasodilator produced by endothelial cells, but also plays an important role in the immune response and infection control [72,73]. In this regard, our data show that the SGE from *Ae. aegypti* reduced NO levels in the serum but increased its production in the peritoneal cavity, the main infectious focus in the CLP model. This effect on NO may be associated with sialloquinins I and II, molecules found in the saliva of *Ae. aegypti* that can induce high NO levels in the host circulation [74]. Sialloquinins are neuropepitides belonging to the tachykinin family, responsible for the vasodilatory action of *Ae. aegypti* saliva [75]. Thus, tachykinins bind to receptors found on the endothelium upregulating iNOS expression through the signaling pathway neurokinin (NK) 1, increasing NO production and ameliorating the vascular capacity [75,76].

In relation to antioxidant action, pre-treatment with SGE from *Ae. aegypti* increased antioxidant capacity and GSH in this sepsis model, showing its protective effect on oxidative damage. In corroboration, a study showed that *Ae. aegypti*’s SGE has protein constituents with antioxidant properties, including SOD, GST, NADH-ubiquinone oxidoreductase, and the electron transporter oxidoreductase [77]. The endogenous GSH acts as a substrate for the enzyme glutathione peroxidase (GPX), which is essential for the detoxification of hydrogen peroxide and lipids [78,79] Under conditions of oxidative stress such as in sepsis, GSH levels can become dramatically depleted [80,81]. Decreased levels of GSH and total antioxidant capacity are directly related to organ failure induced by sepsis and mortality [5]. Thus, the antioxidant compounds from SGE might increase the GSH levels and neutralize the oxidative species. For example, SOD will convert the superoxide into oxygen or hydrogen peroxide that are less reactive molecules, and NADH can restore the oxidized GSSG to its antioxidant form GSH, therefore increasing GSH bioavailability in SGE-pretreated animals.

The lipid peroxidation caused by oxidative damage to membrane lipids, generating lipid free radicals that oxidize other structural lipids, forming a chain reaction that can lead to cell death [82]. MDA is one of the main products of lipid peroxidation and can be used as a biomarker of oxidative stress and cellular damage in diseases related to oxidative stress such as sepsis [83]. In sepsis, MDA levels are elevated and may be correlated with clinical worsening of the patient [84,85]. In our study, the SGE decreased MDA levels in septic animals in all evaluated organs, which can be related to the balance of the oxidative process in the body, mediated by the increase in antioxidant factors and decreased production of ROS and RNS in septic animals. Indeed, Almeras et al. [77] showed that around 10% of the SGE components have oxidoreductase activity [77], which can explain the lower levels of MDA and the increased antioxidant activity in the animals pretreated with SGE.

## 5. Conclusions

**Figure 8:**
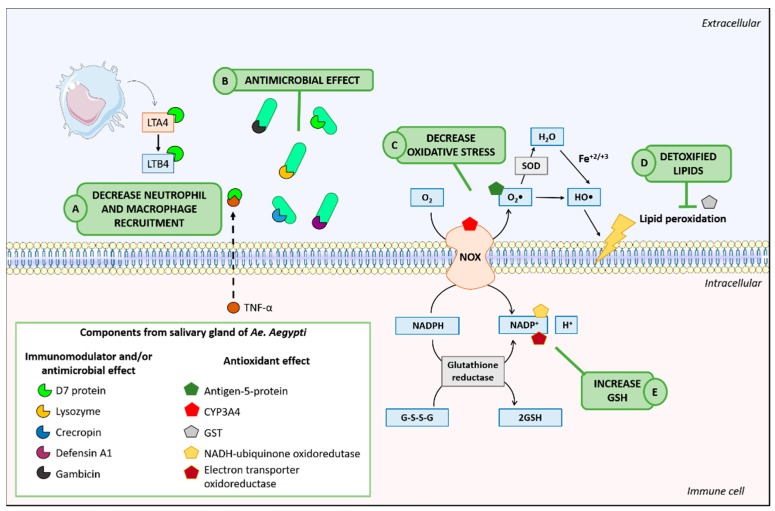
Probable mechanism of action of Ae. aegypti’s SGE in CLP sepsis model. (A) The D7 protein of SGE binds to LTB4 and reduces TNF-α production, decreasing neutrophil and macrophage recruitment to the inflammatory site. (B) The SGE has lysozyme, crecropin, D7 protein, defensin A1, and gambicin, which interact with bacterial surfaces and result their elimination. (C) By a different mechanism, SGE modulates oxidative stress, and SOD converts the superoxide into oxygen or hydrogen peroxide, which are less reactive molecules. (D) SGE reduce lipid peroxidation by restoring antioxidant system. (E) NADH restores the reduced GSH to its active form, increasing GSH bioavailability and reducing lipid peroxidation. CLP: cecal ligation and puncture; SGE: salivary gland extract; LTB4: Leukotriene B4; TNF-α: tumor necrosis factor - alfa; SOD: superoxide dismutase; GSH: glutathione.

In conclusion, our study was the first to show that the pre-treatment with SGE from *Ae. aegypti* led to immunomodulatory and antimicrobial effects on sepsis, increasing survival and reducing bacterial load in septic animals. These data may be associated with the balance between inflammatory and oxidative processes in these animals, as shown in Figure 8. Overall, this study has applied an enthusiastic approach to the prophylactic effect of sepsis throughout the world, but we cannot guarantee that the saliva of *Ae. aegypti* can have a therapeutic effect. However, our data encourage us to investigate the salivary components of *Ae. aegypti*, which may be a new source of drugs for the treatment of different inflammatory diseases in the future. However, other steps are also needed, including finding details on the mechanisms of action, 3D modeling, refinement, and validation, and subsequently molecular and dynamic coupling with possible component receivers, among others.

## Figures and Tables

**Table 1:** Bacterial load in peritoneal lavage, blood and other tissues of septic mice pretreated with SGE (35 µg/kg), CEF (20 mg/kg), or saline.

	Bacterial Load (×10^3^) CFU/mL						
	Groups	Peritoneal Lavage	Blood	Spleen	Heart	Liver	Lung
12 h	Sham	ND	ND	ND	ND	ND	ND
	Saline + CLP	11.5 ± 0.70	4.5 ± 0.70	2.5 ± 0.70	0.5 ± 0.70	3 ± 1.41	19.5 ± 2.12
	CEF + CLP	ND	1 ± 1.41	1.5 ± 0.70	ND	ND	ND
	SGE + CLP	0.54 ± 0.10 ^a^	0.25 ± 0.05 ^a^	0.21 ± 0.10 ^a^	0.05 ± 0.01	0.31 ± 0.05	0.12 ± 0.05 ^a^
24 h	Sham	ND	ND	ND	ND	ND	ND
	Saline + CLP	4 ± 4.24	3.5 ± 2.12	4.5 ± 0.70	3.5 ± 0.70	2 ± 1.41	3 ± 1.41
	CEF + CLP	ND	ND	1.5 ± 0.70	ND	ND	ND
	SGE + CLP	0.005 ± 0.002 ^a^	ND	ND	ND	ND	ND

ND—Not detected ^a^ *p* < 0.05, SGE group + CLP compared to saline group + CLP.

## References

[1] Seymour C.W., Liu V.X., Iwashyna T.J., Brunkhorst F.M., Rea T.D., Scherag A., Rubenfeld G., Kahn J.M., Shankar-Hari M., Singer M. (2016). Assessment of clinical criteria for sepsis for the third international consensus definitions for sepsis and septic shock (sepsis-3). J. Am. Med. Assoc..

[2] Singer M., Deutschman C.S., Seymour C., Shankar-Hari M., Annane D., Bauer M., Bellomo R., Bernard G.R., Chiche J.D., Coopersmith C.M. (2016). The third international consensus definitions for sepsis and septic shock (sepsis-3). J. Am. Med. Assoc..

[3] Ajrouche R., Al-Hajje A., El-Helou N., Awada S., Rachidi S., Zein S., Salameh P. (2013). Statins decrease mortality in Lebanese patients with sepsis: A multicenter study. Pharm. Pract..

[4] Barros L.L.d.S., Maia C.d.S.F., Monteiro M.C. (2016). Fatores de risco associados ao agravamento de sepse em pacientes em Unidade de Terapia Intensiva. Cad. Saúde Coletiva.

[5] Galley H.F. (2011). Oxidative stress and mitochondrial dysfunction in sepsis. Br. J. Anaesth..

[6] Toscano M.G., Ganea D., Gamero A.M. (2011). Cecal Ligation Puncture Procedure. J. Vis. Exp..

[7] Pradipta I.S., Sodik D.C., Lestari K., Parwati I., Halimah E., Diantini A., Abdulah R. (2013). Antibiotic resistance in sepsis patients: Evaluation and recommendation of antibiotic use. N. Am. J. Med. Sci..

[8] Wilson J.X. (2013). Evaluation of Vitamin C for Adjuvant Sepsis Therapy. Antioxid. Redox Signal..

[9] Vogelaers D., De Bels D., Forêt F., Cran S., Gilbert E., Schoonheydt K., Blot S. (2010). Patterns of antimicrobial therapy in severe nosocomial infections: Empiric choices, proportion of appropriate therapy, and adaptation rates-a multicentre, observational survey in critically ill patients. Int. J. Antimicrob. Agents.

[10] Maurya H., Mangal V., Gandhi S., Prabhu K., Ponnudurai K. (2014). Prophylactic antioxidant potential of gallic acid in murine model of sepsis. Int. J. Inflam..

[11] Hara N., Chijiiwa M., Yara M., Ishida Y., Ogiwara Y., Inazu M., Kuroda M., Karlsson M., Sjovall F., Elmér E., Uchino H. (2015). Metabolomic analyses of brain tissue in sepsis induced by cecal ligation reveal specific redox alterations-protective effects of the oxygen radical scavenger edaravone. Shock.

[12] Sales-Campos H., De Souza P.R., Basso P.J., Ramos A.D., Nardini V., Chica J.E.L., Capurro M.L., Sá-Nunes A., De Barros Cardoso C.R. (2015). Aedes aegypti salivary gland extract ameliorates experimental inflammatory bowel disease. Int. Immunopharmacol..

[13] Melo B., Silva N., Gomes R., Navegantes K., Oliveira A., Almeida L., Azevedo C., Monteiro M. (2015). Bioactive Compounds of the Salivary Glands from Aedes aegypti with Anti-Hemostatic Action. Annu. Res. Rev. Biol..

[14] Ribeiro J.M.C., Arcà B., Lombardo F., Calvo E., Phan V.M., Chandra P.K., Wikel S.K. (2007). An annotated catalogue of salivary gland transcripts in the adult female mosquito, *Aedes aegypti*. BMC Genom..

[15] FitzSimmons J., Shah S. (2016). Zika Virus. N. Engl. J. Med..

[16] Monath T.P., Vasconcelos P.F.C. (2015). Yellow fever. J. Clin. Virol..

[17] Chisenhall D.M., Christofferson R.C., McCracken M.K., Johnson A.M.F., Londono-Renteria B., Mores C.N. (2014). Infection with dengue-2 virus alters proteins in naturally expectorated saliva of Aedes aegypti mosquitoes. Parasites Vectors.

[18] Ferreira F.B.D., dos Santos C., Bruxel M.A., Nunes E.A., Spiller F., Rafacho A. (2017). Glucose homeostasis in two degrees of sepsis lethality induced by caecum ligation and puncture in mice. Int. J. Exp. Pathol..

[19] Monteiro M.C., Nogueira L.G., Almeida Souza A.A., Ribeiro J.M.C., Silva J.S., Cunha F.Q. (2005). Effect of salivary gland extract of Leishmania vector, Lutzomyia longipalpis, on leukocyte migration in OVA-induced immune peritonitis. Eur. J. Immunol..

[20] d’Acampora A.J., Locks G.D.F. (2014). Median lethal needle caliber in two models of experimental sepsis. Acta Cir. Bras..

[21] Rittirsch D., Huber-Lang M.S., Flierl M.A., Ward P.A. (2009). Immunodesign of experimental sepsis by cecal lingation and puncture. Nat. Protoc..

[22] Granger D.L., Taintor R.R., Boockvar K.S., Hibbs J.B. (1996). Measurement of nitrate and nitrite in biological samples using nitrate reductase and Griess reaction. Methods Enzymol..

[23] Stuehr D.J., Marletta M.A. (1985). Mammalian nitrate biosynthesis: Mouse macrophages produce nitrite and nitrate in response to Escherichia coli lipopolysaccharide. Proc. Natl. Acad. Sci. USA.

[24] Ferreira-Cravo M., Piedras F.R., Moraes T.B., Ferreira J.L.R., de Freitas D.P.S., Machado M.D., Geracitano L.A., Monserrat J.M. (2007). Antioxidant responses and reactive oxygen species generation in different body regions of the estuarine polychaeta Laeonereis acuta (Nereididae). Chemosphere.

[25] Gomes A., Fernandes E., Lima J.L.F.C. (2005). Fluorescence probes used for detection of reactive oxygen species. J. Biochem. Biophys. Methods.

[26] Miller N.J., Rice-Evans C., Davies M.J., Gopinathan V., Milner A. (1993). A Novel Method for Measuring Antioxidant Capacity and its Application to Monitoring the Antioxidant Status in Premature Neonates. Clin. Sci..

[27] Re R., Pellegrini N., Proteggente A., Pannala A., Yang M., Rice-Evans C. (1999). Antioxidant activity applying an improved ABTS radical cation decolorization assay. Free Radic. Biol. Med..

[28] Ellman G.L. (1959). Tissue sulfhydryl groups. Arch. Biochem. Biophys..

[29] (1944). HENRY IRVING KOHN and Margaret LIVERSEDGE on a New Aerobic Metabolite Whose Production By Brain Is Inhibited By Apomorphine, Emetine, Ergotamine, Epinephrine, and Menadione. J. Pharmacol. Exp. Ther..

[30] Percário S., Vital A.C.C., Jablonka F. (1994). Dosagem do malondialdeido. Newslab.

[31] Ruiz S., Vardon-Bounes F., Merlet-Dupuy V., Conil J.-M., Buléon M., Fourcade O., Tack I., Minville V. (2016). Sepsis modeling in mice: Ligation length is a major severity factor in cecal ligation and puncture. Intensive Care Med. Exp..

[32] Nemzek J.A., Hugunin K.M.S., Opp M.R. (2008). Modeling sepsis in the laboratory: Merging sound science with animal well-being. Comp. Med..

[33] Dejager L., Pinheiro I., Dejonckheere E., Libert C. (2011). Cecal ligation and puncture: The gold standard model for polymicrobial sepsis?. Trends Microbiol..

[34] Song T., Yin H., Chen J., Huang L., Jiang J., He T., Huang H., Hu X. (2016). Survival advantage depends on cecal volume rather than cecal length in a mouse model of cecal ligation and puncture. J. Surg. Res..

[35] Kasuda S., Matsui H., Ono S., Matsunari Y., Nishio K., Shima M., Hatake K., Sugimoto M. (2016). Relevant role of von willebrand factor in neutrophil recruitment in a mouse sepsis model involving cecal ligation and puncture. Haematologica.

[36] Patel A., Joseph J., Periasamy H., Mokale S. (2018). Azithromycin in combination with ceftriaxone reduces systemic inflammation and provides survival benefit in murine model of polymicrobial sepsis. Antimicrob. Agents Chemother..

[37] Schleibinger M., Steinbach C.L., Töpper C., Kratzer A., Liebchen U., Kees F., Salzberger B., Kees M.G. (2015). Protein binding characteristics and pharmacokinetics of ceftriaxone in intensive care unit patients. Br. J. Clin. Pharmacol..

[38] Cuenca A.G., Delano M.J., Kelly-Scumpia K.M., Moldawer L.L., Efron P.A. (2010). Current Protocols in Immunology: Cecal Ligation and Puncture. Curr. Protoc. Immunol..

[39] Echtenacher B., Männel D.N., Hültner L. (1996). Critical protective role of mast cells in a model of acute septic peritonitis. Nature.

[40] Hyde S.R., Stith R.D., McCallum R.E. (1990). Mortality and bacteriology of sepsis following cecal ligation and puncture in aged mice. Infect. Immun..

[41] Gomes R.N., Teixeira-Cunha M.G.A., Figueiredo R.T., Almeida P.E., Alves S.C., Bozza P.T., Bozza F.A., Bozza M.T., Zimmerman G.A., Castro-Faria-Neto H.C. (2013). Bacterial clearance in septic mice is modulated by MCP-1/CCL2 and nitric oxide. Shock.

[42] Calvo E., Mans B.J., Ribeiro J.M.C., Andersen J.F. (2009). Multifunctionality and mechanism of ligand binding in a mosquito antiinflammatory protein. Proc. Natl. Acad. Sci. USA.

[43] Ribeiro J.M.C., Martin-Martin I., Arcá B., Calvo E. (2016). A deep insight into the sialome of male and female aedes aegypti mosquitoes. PLoS ONE.

[44] Wichit S., Ferraris P., Choumet V., Missé D. (2016). The effects of mosquito saliva on dengue virus infectivity in humans. Curr. Opin. Virol..

[45] Assumpção T.C.F., Ma D., Schwarz A., Reiter K., Santana J.M., Andersen J.F., Ribeiro J.M.C., Nardone G., Yu L.L., Francischetti I.M.B. (2013). Salivary antigen-5/CAP Family members are Cu2+-dependent antioxidant enzymes that scavenge O2.- and inhibit collagen-induced platelet aggregation and neutrophil oxidative burst. J. Biol. Chem..

[46] Rossignol P.A., Lueders A.M. (1986). Bacteriolytic factor in the salivary glands of Aedes aegypti. Comp. Biochem. Physiol. Part B Biochem..

[47] Juhn J., Naeem-Ullah U., MacIel Guedes B.A., Majid A., Coleman J., Paolucci Pimenta P.F., Akram W., James A.A., Marinotti O. (2011). Spatial mapping of gene expression in the salivary glands of the dengue vector mosquito, aedes aegypti. Parasites Vectors.

[48] Sim S., Ramirez J.L., Dimopoulos G. (2012). Dengue virus infection of the aedes aegypti salivary gland and chemosensory apparatus induces genes that modulate infection and blood-feeding behavior. PLoS Pathog..

[49] Zhang R., Zhu Y., Pang X., Xiao X., Zhang R., Cheng G. (2017). Regulation of Antimicrobial Peptides in Aedes aegypti Aag2 Cells. Front. Cell. Infect. Microbiol..

[50] Vizioli J., Bulet P., Hoffmann J.A., Kafatos F.C., Muller H.-M., Dimopoulos G. (2001). Gambicin: A novel immune responsive antimicrobial peptide from the malaria vector Anopheles gambiae. Proc. Natl. Acad. Sci. USA.

[51] Lai Y., Gallo R.L. (2009). AMPed up immunity: How antimicrobial peptides have multiple roles in immune defense. Trends Immunol..

[52] Warr E., Das S., Dong Y., Dimopoulos G. (2008). The Gram-Negative Bacteria-Binding Protein gene family: Its role in the innate immune system of Anopheles gambiae and in anti-Plasmodium defence. Insect Mol. Biol..

[53] Barroqueiro E.S.B., Prado D.S., Barcellos P.S., Silva T.A., Pereira W.S., Silva L.A., Maciel M.C.G., Barroqueiro R.B., Nascimento F.R.F., Gonçalves A.G. (2016). Immunomodulatory and Antimicrobial Activity of Babassu Mesocarp Improves the Survival in Lethal Sepsis. Evidence Based Complement. Altern. Med..

[54] Kobayashi M., Nakamura K., Cornforth M., Suzuki F. (2012). Role of M2b Macrophages in the Acceleration of Bacterial Translocation and Subsequent Sepsis in Mice Exposed to Whole Body [137Cs] Gamma-Irradiation. J. Immunol..

[55] Xiao H., Siddiqui J., Remick D.G. (2006). Mechanisms of mortality in early and late sepsis. Infect. Immun..

[56] Navegantes K.C., de Souza Gomes R., Pereira P.A.T., Czaikoski P.G., Azevedo C.H.M., Monteiro M.C. (2017). Immune modulation of some autoimmune diseases: The critical role of macrophages and neutrophils in the innate and adaptive immunity. J. Transl. Med..

[57] De Oliveira S., Rosowski E.E., Huttenlocher A. (2016). Neutrophil migration in infection and wound repair: Going forward in reverse. Nat. Rev. Immunol..

[58] Li P., Oh D.Y., Bandyopadhyay G., Lagakos W.S., Talukdar S., Osborn O., Johnson A., Chung H., Mayoral R., Maris M. (2015). LTB4 promotes insulin resistance in obese mice by acting on macrophages, hepatocytes and myocytes. Nat. Med..

[59] Coutinho-Abreu I.V., Guimarães-Costa A.B., Valenzuela J.G. (2015). Impact of insect salivary proteins in blood feeding, host immunity, disease, and in the development of biomarkers for vector exposure. Curr. Opin. Insect Sci..

[60] Schneider B.S., Soong L., Zeidner N.S., Higgs S. (2004). *Aedes aegypti* salivary gland extracts modulate anti-viral and TH1/TH2 cytokine responses to sindbis virus infection. Viral Immunol..

[61] Calvo E., Tokumasu F., Marinotti O., Villeval J.L., Ribeiro J.M.C., Francischetti I.M.B. (2007). Aegyptin, a novel mosquito salivary gland protein, specifically binds to collagen and prevents its interaction with platelet glycoprotein VI, integrin α2β1, and von Willebrand factor. J. Biol. Chem..

[62] Schneider B.S., Soong L., Coffey L.L., Stevenson H.L., McGee C.E., Higgs S. (2010). Aedes aegypti Saliva Alters Leukocyte Recruitment and Cytokine Signaling by Antigen-Presenting Cells during West Nile Virus Infection. PLoS ONE.

[63] Monteiro M.C., Lima H.C., Souza A.A.A., Titus R.G., Romão P.R.T., Cunha F.D.Q. (2007). Effect of Lutzomyia longipalpis salivary gland extracts on leukocyte migration induced by Leishmania major. Am. J. Trop. Med. Hyg..

[64] Saeaue L., Morales N.P., Komalamisra N., Vargas R.E.M. (2011). Antioxidative systems defense against oxidative stress induced by blood meal in Aedes aegypti. Southeast Asian J. Trop. Med. Public Health.

[65] Oliveira J.H.M., Talyuli O.A.C., Goncalves R.L.S., Paiva-Silva G.O., Sorgine M.H.F., Alvarenga P.H., Oliveira P.L. (2017). Catalase protects Aedes aegypti from oxidative stress and increases midgut infection prevalence of Dengue but not Zika. PLoS Negl. Trop. Dis..

[66] Ness T.L., Hogaboam C.M., Strieter R.M., Kunkel S.L. (2003). Immunomodulatory Role of CXCR2 During Experimental Septic Peritonitis. J. Immunol..

[67] Sônego F., Castanheira F.V.e.S., Ferreira R.G., Kanashiro A., Leite C.A.V.G., Nascimento D.C., Colón D.F., Borges V.d.F., Alves-Filho J.C., Cunha F.Q. (2016). Paradoxical roles of the neutrophil in sepsis: Protective and deleterious. Front. Immunol..

[68] Craciun F.L., Schuller E.R., Remick D.G. (2010). Early Enhanced Local Neutrophil Recruitment in Peritonitis-Induced Sepsis Improves Bacterial Clearance and Survival. J. Immunol..

[69] Wang X., Qin W., Qiu X., Cao J., Liu D., Sun B. (2014). A novel role of exogenous carbon monoxide on protecting cardiac function and improving survival against sepsis via mitochondrial energetic metabolism pathway. Int. J. Biol. Sci..

[70] Cimolai M.C., Alvarez S., Bode C., Bugger H. (2015). Mitochondrial mechanisms in septic cardiomyopathy. Int. J. Mol. Sci..

[71] Förstermann U., Sessa W.C. (2012). Nitric oxide synthases: Regulation and function. Eur. Heart J..

[72] Victor V.M., Rocha M., Esplugues J.V., De la Fuente M. (2005). Role of Free Radicals in Sepsis: Antioxidant Therapy. Curr. Pharm. Des..

[73] Bogdan C. (2015). Nitric oxide synthase in innate and adaptive immunity: An update. Trends Immunol..

[74] Champagne D.E., Ribeiro J.M. (1994). Sialokinin I and II: Vasodilatory tachykinins from the yellow fever mosquito Aedes aegypti. Proc. Natl. Acad. Sci. USA.

[75] Ribeiro J.M.C. (1992). Characterization of a vasodilator from the salivary glands of the yellow fever mosquito Aedes aegypti. J. Exp. Biol..

[76] Mulè F., Baffi M.C., Capparelli A., Pizzuti R. (2003). Involvement of nitric oxide and tachykinins in the effects induced by protease-activated receptors in rat colon longitudinal muscle. Br. J. Pharmacol..

[77] Almeras L., Fontaine A., Belghazi M., Bourdon S., Boucomont-Chapeaublanc E., Orlandi-Pradines E., Baragatti M., Corre-Catelin N., Reiter P., Pradines B. (2010). Salivary gland protein repertoire from Aedes aegypti mosquitoes. Vector Borne Zoonotic Dis..

[78] Forman H.J., Zhang H., Rinna A. (2009). Glutathione: Overview of its protective roles, measurement, and biosynthesis. Mol. Aspects Med..

[79] Biolo G., Antonione R., De Cicco M. (2007). Glutathione metabolism in sepsis. Crit. Care Med..

[80] Albuszies G., Albuszies G., Brückner U.B. (2003). Antioxidant therapy in sepsis. Intensive Care Med.

[81] Prauchner C.A. (2017). Oxidative stress in sepsis: Pathophysiological implications justifying antioxidant co-therapy. Burns.

[82] Goode H.F., Cowley H.C., Walker B.E., Howdle P.D., Webster N.R. (1995). Decreased antioxidant status and increased lipid peroxidation in patients with septic shock and secondary organ dysfunction. Crit. Care Med..

[83] Weiss S.L., Deutschman C.S. (2014). Elevated malondialdehyde levels in sepsis - something to “stress” about?. Crit. Care.

[84] Garrabou G., Morén C., López S., Tobías E., Cardellach F., Miró Ò., Casademont J. (2012). The effects of sepsis on mitochondria. J. Infect. Dis..

[85] Şener G., Toklu H., Kapucu C., Ercan F., Erkanli G., Kaçmaz A., Tilki M., Yeǧen B.Ç. (2005). Melatonin protects against oxidative organ injury in a rat model of sepsis. Surg. Today.

